# Audience Response System Facilitates Prediction of Scores on In-Training Examination

**DOI:** 10.5811/westjem.2017.1.32858

**Published:** 2017-03-03

**Authors:** Kaushal H. Shah, Jaime Jordan, Katherine Jahnes, David P. Lisbon, Lucienne Lutfy-Clayton, Grant Wei, Gary Winkel, Sally A. Santen

**Affiliations:** * Icahn School of Medicine at Mt. Sinai, Department of Emergency Medicine, New York, New York; † Harbor-UCLA Medical Center, Department of Emergency Medicine, Torrance, California; ‡ NYU Langone Medical Center, Ronald O. Perelman Department of Emergency Medicine, New York, New York; § University of Kansas Hospital, Department of Emergency Medicine, Kansas City, Kansas; ¶ University of Massachusetts Medical School - Baystate Health, Department of Emergency Medicine, Springfield, Massachusetts; || Robert Wood Johnson University Hospital, Department of Emergency Medicine, New Brunswick, New Jersey; # University of Michigan, Department of Emergency Medicine, Ann Arbor, Michigan

## Abstract

**Introduction:**

Audience response systems (ARS) are increasingly popular; however, their contribution to education is not completely clear. Our study found that scores from review quizzes delivered by an ARS correlate with in-training exam (ITE) scores and are viewed positively by residents. This information may be useful in identifying poor performers early so that targeted educational interventions can be made. The objective was to determine if scores on review quizzes delivered by an ARS correlate with ITE scores and to obtain participant feedback on use of the ARS for ITE preparation.

**Methods:**

This was a prospective observational study of emergency medicine (EM) residents at six accredited EM residency programs. Subjects included residents who had taken previous ITEs. Subjects participated in bimonthly review sessions using an ARS. Twelve review quizzes were administered, each consisting of 10 multiple-choice questions. After the ITE, subjects completed an attitudinal survey consisting of six Likert-scale items and one “yes/no” item. We used a mixed linear model to analyze the data, accounting for prior 2012 ITE scores and nesting due to institution.

**Results:**

Among 192 participants, 135 (70.3%) completed the ITE in both 2012 and 2013; we analyzed their data for the first objective. Results from the mixed linear model indicate that the total mean score on the review quizzes was a significant [t(127) = 6.68; p < 0.001] predictor of the 2013 ITE after controlling for the 2012 ITE score. One hundred forty-six (76.0%) participants completed the attitudinal survey; 96% of respondents stated that they would like ARS to be used more often in resident education. Respondents felt the sessions aided in learning (mean 7.7/10), assisted in preparation for the ITE (mean 6.7/10), and helped identify content areas of weakness (mean 7.6/10).

**Conclusion:**

Our results suggest that scores from review quizzes delivered by an audience response system correlate with in-training exam scores and is viewed positively by residents.

## INTRODUCTION

To become board certified, emergency physicians must pass the American Board of Emergency Medicine (ABEM) written and oral certification examinations. In preparation, residents take an annual in-training exam (ITE). EM residencies aim for all residents to pass the written examination. As a result, it is common practice for residencies to dedicate specific didactic time as review sessions to improve ITE scores. The amount and method of this preparation is variable. We sought to develop a curriculum using an audience response system (ARS) that could potentially predict how residents would perform on the ITE.

There are a number of ARSs available and they are increasingly used for didactic teaching. They involve wireless technology where participants send a response via keypads, clickers or cell phones to a computer that then tallies and projects those responses to the audience. The audience responses to questions or stimuli can be embedded graphically in a PowerPoint lecture providing immediate feedback to the audience about their input. ARS have been shown to improve the effectiveness of didactic lectures by increasing attendance, attention levels, motivation, participation and engagement.^1^–^8^

The literature on ARS is clear that students embrace this technology as a learning tool; however, it remains unclear whether participation and tracking of results through an ARS can assist educators in predicting which students will do poorly on an annual comprehensive exam. If such a system could predict those at risk for poor outcomes, early targeted educational interventions could take place. The primary objective of this study was to determine if the results of bi-monthly, written, board-style questions using the ARS were correlated with the annual ITE scores among EM residents from six different programs. In addition, we wanted to determine resident reactions to the use of an ARS for ITE review.

**Population Health Research Capsule**

What do we already know about this issue?

Learners enjoy the use of an audience response system for didactic education. Whether it is valuable in predicting or improving learning is not entirely clear.

What was the research question?

Do scores on review quizzes delivered in resident conference using an audience response system correlate with scores on the in-training exam?

What was the major finding of the study?

Review quizzes delivered by an audience response system are viewed positively by residents, and results correlate with in-training exam scores.

How does this improve population health?

Improvements in the education of core content to residents will likely improve the quality of care delivered by them in the long run.

## METHODS

### Study Setting and Population

Residents from six EM residency programs accredited by the Accreditation Council for Graduate Medical Education participated in this study. Collaboration was facilitated through the MERC (Medical Education Research Certificate) at CORD (Council of Residency Directors) Program. The table describes the residency programs involved. Written informed consent was obtained from each subject prior to initiation of the study. The institutional review board of each institution approved the study protocol.

### Study Design

This prospective, multicenter cohort study was conducted from August 2012 to January 2013. Study participants were all the EM residents at each training site that routinely attend conference. They were all consented for participation. No residents were excluded; however, because first-year residents at Harbor-UCLA Medical Center do not attend didactic conference they were not consented and they did not participate. Although the interns at the other sites did not have prior ITE scores for comparison, they were included in the study because they would be participating in the ARS and completing the post-survey. All participants present for the session answered EM board-style questions during didactic conference twice per month for the six months (total 12 sessions) preceding the ITE in February 2013. Each program has regular didactics that occur on a weekly basis. Residents who were present for the board review (which could vary from week to week due to clinical responsibilities that prevent attendance, e.g., working in the intensive care unit or scheduled to work the night before conference) would voluntarily answer the questions via the audience response clickers. Administration of questions was done by a single person at each institution, using the Turning Point Technology^™^ ARS.

Topics were chosen a priori based on the list of most commonly asked question topics published by ABEM on their website.^9^ However, the residents were not aware of topics prior to the session. The 12 topics are listed in Appendix 1. Each review session consisted of 10 questions on a particular topic that were randomly obtained from a question bank created by emergency physicians developing what is now RoshReview, LLC. The questions were developed primarily for a novel, web-based question bank for resident preparation for the national ABEM-certifying exam. When this study was designed and initiated, these questions were not released to the public. Since the questions were not available to the study participants, they were ideal because residents could not have prior knowledge of the correct answers.

Turning Point Technologies^™^ (Youngstown, Ohio) is a specific audience response product using audience clickers, which send feedback to a receiver with a USB hub that attaches to the computer. It is completely integrated with PowerPoint such that the slideshow appears identical to what residents are accustomed to seeing. Subjects were given a question and multiple-answer choices on the slide. After everyone clicked their answer selection, a graphic display of the percentage or number of subjects who selected each choice was displayed for everyone to see. Participants were not individually identified. Thereafter, a checkmark appeared informing the audience of the correct answer. There wasn’t a scripted discussion of the correct answers, but the administrator of the questions was allowed to explain why the answer was correct and why the other options were incorrect.

### Study Protocol

One investigator (KS) randomly selected the 10 questions for each topic from the topic-specific pool of questions on the RoshReview website. Questions were then placed in a PowerPoint presentation that allowed for use with Turning Point Technologies^™^. PowerPoint sessions were then sent to the lead investigator at each site. Sessions were consistently administered by the lead investigators (DL, GW, JJ, KJ, KS, LLC) twice a month for six months to cover the 12 most commonly tested topics. If a session could not be administered in the assigned month, the topic was skipped to ensure all participants completed the questions at the same time in their residency training.

Residents were assigned particular clickers that they used for each session, thereby maintaining a unique identifier that remained de-identified to the study investigators. The answer choice selected by each participant (and correct or incorrect designation) was automatically recorded with the unique identifier of each participant. At the completion of each session, data automatically generated by the ARS was sent to one investigator (KS) for collection.

After the ITE, subjects completed a questionnaire to determine their attitudes toward the review sessions delivered by an ARS. The questionnaire consisted of six 10-point Likert-type items and one “yes/no” item. The questions were developed by the research group with attention to content validity through iterative drafts of the survey. Internal structure and response process validity was supported by adherence to survey design principles, review by an educational research expert, and piloting and revision of survey; consistency was determined by Crohnbach’s alpha of 0.81. See list of questions in Appendix 2.

### Data Analysis

To control for prior performance on the ITE, we completed this analysis using only residents who had a 2012 ITE score in addition to a 2013 ITE score. Analysis was performed using a nested mixed linear regression model using SAS version 9.3. We calculated the score on each quiz in terms of the percentage correct, and adjusted the total percentage correct on all tests by the total number of tests taken. Scores from incomplete quizzes, defined as less than 7 out of 10 questions answered, were excluded from analysis. The total ITE score in 2013 was the outcome and it was adjusted for each participant’s 2012 ITE score and the participant’s institution. We included all available demographic variables and the institution in the model. For the attitudinal responses, mean ratings with standard deviations for Likert scale items were calculated using Excel. Response rate for the single “yes/no” question was also recorded.

## RESULTS

A total of 192 residents participated in the study. We included only 135 participants in the primary analysis because 57 participants did not have both a 2012 and 2013 ITE score. Results from the mixed linear model indicate that the total mean score on the review quizzes was a significant [t(127) = 6.68; *p* < 0.0001] predictor of the 2013 ITE score after controlling for the 2012 ITE score.

One hundred forty-six participants (76.0%) completed the survey evaluation of the ARS. Of the 146 residents who completed the attitudinal survey, 95.8% (140) stated “yes” they would like ARS to be used more often in resident education. Participants overall enjoyed the ARS review sessions with a mean score of 8.7 **±** 1.8 on a 10-point scale. They also felt that these sessions aided in learning (mean 7.7 **±** 1.8), assisted in preparation for the ITE (mean 6.7 **±** 2.1), and helped identify content areas of weakness (mean 7.6 **±** 2.0). Participants were equivalent on whether the ARS review sessions prompted them to study more (mean 5.8 **±** 2.7).

## DISCUSSION

This study found a positive correlation between total mean scores on review quizzes delivered by an ARS and ITE scores, after controlling for prior ITE score. These results suggest that review quiz scores may be predictive of ITE scores. Many programs use various forms of “practice tests” or “quizzes” as preparation, but there is little published data in EM to suggest that performance on these tests or quizzes can predict ITE scores.

Residents who have done poorly on the ITE are often encouraged or required to complete some form of remediation or targeted educational intervention; this can improve future outcomes.^10^ However, it is late in their first year of training that residents have completed the ITE and receive their score. Our study suggests that review quizzes delivered by an ARS can be used to help identify residents at risk of poor test outcomes earlier in their course. This is valuable information for residents, program directors and physician educators.

Consistent with prior research on ARS, participants in our study provided positive feedback about this type of educational intervention. This is also not surprising as an ARS allows for increased interactivity and active learning, which are both enjoyable to learners and can positively impact outcomes.^11^,^12^ This may also be a reflection of learner preferences, as active methods have been recommended for “millennial learners.”^13^

In addition to being engaging and stimulating, the ARS when used for ITE preparation or core content knowledge acquisition for residents has two additional features that are important specifically for group testing, including anonymity and self-assessment. It is clear based on many reports that students value anonymity;^14^–^21^ the likely reason is that it eliminates the fear of being judged by peers and instructors. By eliminating this fear, more students will likely attempt to recall and grapple with the content of the material, which can lead to greater participation and greater understanding. In fact, anonymity of clicker responses likely increases responses from students who do not normally respond when general participation is requested.^22^ Using an ARS helps improve the feedback process by allowing anonymity, immediately collecting and summarizing student responses, and preventing participants from copying the answers from their peers.

Displaying all responses also allows learners to gauge their performance against the group, a critical feature for ITE preparation. There is some evidence to suggest that students like to know how well they are performing relative to their peers.^14^,^15^,^19^,^23^,^24^ Students may want to monitor their progress or seek assurance that they are not alone in their misunderstanding of key concepts. If you’re among a small group to choose the wrong answer (weaker knowledge base), the self-assessment is very different than when multiple wrong choices were selected by the group (difficult question). In fact, resident participants noted that the ARS review sessions helped them identify areas of weakness.

This information will significantly contribute to the current body of knowledge in that we have found a potential predictor of ITE scores in a method that trainees view positively^3^,^25^ and may increase their learning.^5^,^6^,^26^,^27^ This method can also assist residents and their residency educators in preparation for the ITE by identifying areas of weakness.

## LIMITATIONS

This study has several limitations. Although there are a large number of participants in our study, the number of questions in each session (10) was small. A greater number of questions/topics would likely more accurately stratify resident knowledge base. RoshReview questions that we used do not have validity evidence. It is unclear if these questions correspond accurately to the ITE material. However, the authors, who are all leaders in EM education, provided content validity in the questions used in the review sessions, although item analysis on the questions was not performed.

Three of the study sites did not complete all the scheduled quizzes. There were logistical issues with conference scheduling and technical difficulties that prevented site investigators from completing the ARS quizzes within the designated month. Although this is a real limitation of the study, given that there were six sites, multiple sessions and multiple questions, we don’t believe the analysis or study outcome was compromised.

We chose to study the ARS as a potential predictor of ITE scores, but certainly paper quizzes or independent computer quizzes with immediate feedback could similarly correlate with performance. Comparing the various evaluation modalities is certainly an area of future research.

Finally, as this study only looked at mean total scores across multiple months and quizzes (12 quizzes over six months), we do not know the minimum number of ARS quiz scores necessary (e.g., are three quizzes enough?) that are correlated with higher ITE scores. This is an area that requires future research.

## CONCLUSION

Performance on review quizzes delivered by an audience response system is correlated with resident in-training exam scores. This type of review is viewed positively by residents and can assist residents in identifying areas of weakness and preparing for the in-training exam.

## Supplementary Information





## Figures and Tables

**Table:** Characteristics of participating institutions at time of a study of the effect of an audience response system on emergency medicine residents’ in-service exam scores.

Residency program	Years of Post-graduate training	Number of residents in program	Resident male/female ratio	Average age (SD)	Number of sessions completed
Mt. Sinai	4	60	35:25	29.0 (2.1)	12
Baystate	3	38	24:14	NA	7
Rutgers Robert Wood Johnson	3	16	11:5	29.1 (2.7)	9
NY Methodist	3	30	16:14	29.9 (2.6)	12
Harbor-UCLA	3	45*	16:14	29.3 (2.7)	12
University of Kansas	3	18	12:6	30.0 (4.0)	8

*NA,* not available.

* Only 30 residents were eligible to participate because interns do not typically attend conference at this training program.
