# Supplementary material for: Audience Response System Facilitates Prediction of Scores on In-Training Examination
Source: West J Emerg Med. 2017 Mar 3;18(3):525–30. doi: 10.5811/westjem.2017.1.32858 (PMC5391905; doi:10.5811/westjem.2017.1.32858)
Supplement: Supplementary file 1 [file wjem-18-525-s001.pdf]

Appendix Table 1: List of topics reviewed over 6 months prior to the ITE

**Topic Reviewed**	**Dates**
Cardiovascular	August
Gastrointestinal	August
Neurology	September
Toxicology	September
Signs & Symptoms	October
Procedures	October
Trauma	November
Respiratory	November
Obstetric/gynecology	December
Infectious Disease	December
HEENT	January
Environmental	January
