# Supplementary material for: Audience Response System Facilitates Prediction of Scores on In-Training Examination
Source: West J Emerg Med. 2017 Mar 3;18(3):525–30. doi: 10.5811/westjem.2017.1.32858 (PMC5391905; doi:10.5811/westjem.2017.1.32858)
Supplement: Supplementary file 2 [file wjem-18-525-s002.pdf]

APPENDIX B – Table 2

Attitudinal Survey Regarding Audience Response System Use*

1. I enjoyed the bi-monthly board review questions delivered by the audience response system.	1 =strongly disagree<br>10 =strongly agree
2. How much did you learn from the bi-monthly board review questions?	1 =Nothing<br>10 =A lot
3. How much did the board review questions by ARS assist in your preparation for the in-training exam?	1 =Not at all<br>10 =A lot
4. How much did the immediate feedback of the ARS on board review question help you identify areas of weakness?	1 =Not at all<br>10 =A lot
5. To what extent did the immediate feedback of the ARS on board review questions prompt you to study more?	1 =Not at all<br>10 =A lot
6. Would you like the ARS to be used more often in resident education?	Yes/No

* Cronbach’s Alpha was 0.81
